# Application of Whole-Exome Sequencing in Identifying the Molecular Basis of Idiopathic Male Infertility

**DOI:** 10.3390/jcm15187281

**Published:** 2026-09-19

**Authors:** Filip Koszałka, Aleksandra Gałan, Oliwier Bułdak, Małgorzata Świąder, Jagoda Góra, Iga Kuliniec, Izabela Zakrocka, Przemysław Mitura, Wojciech Załuska

**Affiliations:** 1 Students’ Scientific Club, Department of Urology and Urological Oncology, Medical University of Lublin, Jaczewskiego 8, 20-090 Lublin, Poland; filipkoszalka6@outlook.com (F.K.); aleksandra.galan00@gmail.com (A.G.); jagodaa1436@gmail.com (J.G.); 2 Students’ Scientific Club of Nephrology and Transplantology, Medical University of Lublin, Jaczewskiego 8, 20-090 Lublin, Poland; buldakoliwier@gmail.com (O.B.); m.swiader27@gmail.com (M.Ś.); 3 Department of Urology and Urological Oncology, Medical University of Lublin, Jaczewskiego 8, 20-090 Lublin, Poland; iga.kuliniec@umlub.edu.pl (I.K.); przemyslaw.mitura@umlub.edu.pl (P.M.); 4 Department of Nephrology, Medical University of Lublin, Jaczewskiego 8, 20-090 Lublin, Poland; wojciech.zaluska@umlub.edu.pl

**Keywords:** male infertility, idiopathic male infertility, whole-exome sequencing, non-obstructive azoospermia, genetic diagnostics, pathogenic variants, variants of uncertain significance, spermatogenic failure

## Abstract

Male infertility represents a major clinical challenge. Despite standard genetic testing (karyotyping, Y-chromosome azoospermia factor (AZF) microdeletion testing, and CFTR variant analysis), the molecular cause remains unidentified in a substantial proportion of patients. These patients are consequently diagnosed with idiopathic male infertility. This review provides a comprehensive overview of the current evidence regarding the application of whole-exome sequencing (WES) in the molecular diagnosis and clinical management of idiopathic male infertility. WES can identify pathogenic variants associated with quantitative spermatogenic defects, qualitative abnormalities of sperm motility and morphology, and pre-testicular causes related to hypogonadotropic hypogonadism. When defining the cohort strictly as patients with true idiopathic non-obstructive azoospermia (NOA) who remain undiagnosed after standard testing, the pooled diagnostic yield of WES is approximately 10–15%. Estimates vary due to differences in cohort selection, variant interpretation, and the range of genes analysed. Establishing a precise molecular diagnosis improves genetic counselling, helps predict the likelihood of successful sperm retrieval via testicular sperm extraction (TESE) or microdissection TESE (micro-TESE), and informs treatment planning for assisted reproductive technologies. However, routine clinical implementation remains limited by the high frequency of variants of uncertain significance, the absence of standardised diagnostic pipelines, unequal access to testing, and ethical concerns. Emerging “all-in-one” diagnostic strategies, multi-omics integration, and whole-genome or long-read sequencing hold promise for improving genomic diagnostics. However, broader adoption will ultimately require ongoing standardisation and functional validation of identified variants.

## 1. Introduction

Infertility is a major global health problem estimated to affect around 17.5% of people of reproductive age [1]. Male factors are involved in approximately 50% of cases, either alone or in combination with female factors [2,3,4]. Male infertility is phenotypically heterogeneous and may present as a quantitative abnormality, such as oligozoospermia or azoospermia, or as a qualitative defect affecting sperm motility or morphology, termed asthenozoospermia and teratozoospermia, respectively [3].

However, it is not just a reproductive issue; it carries severe psychological and social burdens. Patients often experience profound psychological distress and social stigma, leading to a diminished quality of life. In men, a diagnosis of infertility may contribute to social isolation and low self-esteem and is associated with an increased risk of anxiety, depression, and sexual dysfunction [5]. Infertile men may also have an increased risk of certain malignancies [2,3,6]. Current evidence supports a shared genetic aetiology, suggesting that variants responsible for impaired spermatogenesis may also increase susceptibility to tumourigenesis, although a direct causal chain linking spermatogenic variants to cancer development has not yet been firmly established [3,6].

Standard genetic testing, comprising karyotype analysis, Y-chromosome azoospermia factor (AZF) microdeletion testing, and *CFTR* variants analysis, identifies the cause in only a minority of patients [6,7]. Consequently, approximately 40% of cases remain without a precise aetiological diagnosis and are classified as idiopathic [6]. The authoritative systematic review by the International Male Infertility Genomics Consortium (IMIGC) explicitly classified 120 genes with moderate, strong, or definitive evidence of association with 104 male infertility phenotypes, a catalogue that continues to expand with ongoing curation [8]. Addressing this diagnostic gap by identifying the monogenic causes using whole-exome sequencing (WES) has therefore become a major research priority in reproductive medicine [3,4,6,7].

## 2. Literature Search Strategy

To ensure methodological transparency while maintaining the format of a narrative review, we conducted a targeted literature search. We queried the PubMed database and Google Scholar, focusing primarily on articles published between 2020 and 2026, with the search started in May 2026 and finalised in July 2026. While the review of clinical WES diagnostic yields and guidelines focused on this recent timeframe, foundational primary literature was included regardless of publication date. The search strategy utilised combinations of terms including “male infertility”, “whole-exome sequencing”, “WES”, “non-obstructive azoospermia”, “teratozoospermia”, and “monogenic causes”. Articles were included if they provided original genomic data, allowed for the synthesis of overall and phenotype-specific WES diagnostic yields (e.g., for NOA, OA, and teratozoospermia), investigated relevant functional validations, or offered systematic evidence synthesis (e.g., gene–disease validity curation or clinical practice guidelines) informing the review’s conclusions. Studies focusing exclusively on female infertility or purely environmental factors were excluded. In addition, the quality of this narrative review was self-assessed using the Scale for the Assessment of Narrative Review Articles (SANRA) checklist (Document S1). 

## 3. Aetiology of Male Infertility: Classification

Clinically, male infertility is viewed through two lenses: anatomical-functional and genetic-environmental. The anatomical–functional classification distinguishes obstructive azoospermia (OA), caused by obstruction of the seminal outflow tract despite preserved spermatogenesis, from non-obstructive azoospermia (NOA), in which the primary defect affects spermatogenesis within the seminiferous tubules. NOA may be further divided into pre-testicular causes associated with dysfunction of the hypothalamic–pituitary–gonadal axis and testicular causes arising from primary damage to gonadal tissue [9].

The genetic–environmental classification describes the nature of the causative factor. Genetic causes include chromosomal abnormalities, Y-chromosome AZF microdeletions, single-gene variants, and epigenetic alterations affecting the expression of genes involved in spermatogenesis [10,11]. Environmental contributors include exposure to toxins, increased scrotal temperature, radiation, endocrine-disrupting chemicals, smoking, and obesity. Genetic and environmental factors rarely act independently; the infertility phenotype commonly reflects their interaction [11].

Current guidelines from the American Urological Association and the American Society for Reproductive Medicine (AUA/ASRM) recommend targeted genetic testing, including karyotype and AZF microdeletion analysis, primarily for patients with azoospermia or severe oligozoospermia. Routine detection of genetic causes is therefore restricted to a narrowly defined group of patients [12]. Men in whom imaging, hormonal assessment, and initial genetic testing do not identify a cause are diagnosed with idiopathic infertility [10]. Idiopathic infertility is, however, not a distinct disease; it simply highlights the limits of current diagnostic testing. A proportion of these patients are likely to have an undetected monogenic cause that is not captured by standard tests [7,10]. They therefore represent the principal population in which the diagnostic use of WES may be considered.

## 4. Limitations of Standard Genetic Diagnostics

The standard genetic testing pathway for men with severe fertility disorders comprises karyotype analysis, Y-chromosome AZF microdeletion testing, and *CFTR* variants analysis (Table 1). According to the current AUA/ASRM guidelines, karyotyping is indicated in men with azoospermia or a sperm concentration below 5 million/mL when accompanied by elevated concentration of follicle-stimulating hormone (FSH), testicular atrophy, or evidence of impaired spermatogenesis. AZF microdeletion analysis is indicated in men with azoospermia or a sperm concentration of ≤1 million/mL under similar clinical circumstances [12]. *CFTR* testing is recommended for men with congenital absence of the vas deferens or idiopathic obstructive azoospermia [3,12]. These eligibility criteria restrict standard genetic testing to a relatively small group of severely affected patients.

Even among eligible patients, the combined diagnostic yield of these tests remains limited. Routinely investigated genetic abnormalities are estimated to explain only 4–9.2% of all cases of male infertility. In NOA, where the genetic contribution is particularly high, standard testing identifies the cause in no more than approximately 25% of cases [7]. Each test also has a restricted scope. Karyotyping detects numerical and structural chromosomal abnormalities, AZF testing examines three defined regions of the long arm of the Y chromosome, and *CFTR* analysis evaluates a single gene associated primarily with obstructive infertility caused by congenital absence of the vas deferens [3,12]. These tests do not detect variants in the many other genes with confirmed or probable associations with abnormalities of spermatogenesis, sperm motility, or morphology. The number of such genes continues to increase with advances in next-generation sequencing (NGS) [3,4].

**Figure 1:**
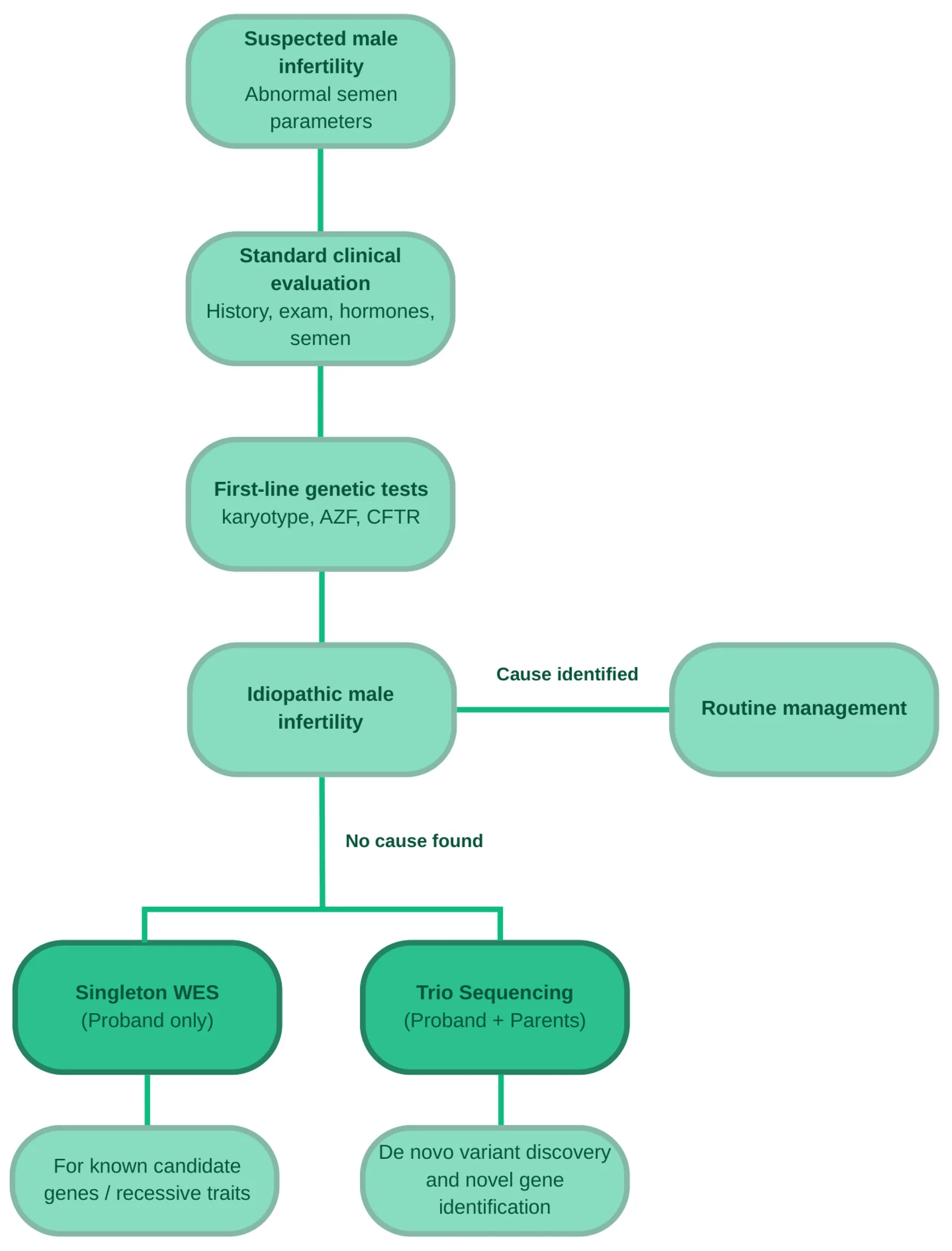
Proposed diagnostic algorithm based on genetic tests in infertile males. Figure created using Canva Pro (Canva Pty Ltd., Sydney, Australia; https://www.canva.com).

In many men with abnormal semen parameters and normal standard genetic findings, the molecular cause remains unidentified because the available tests were not designed to detect the full range of monogenic disorders [4,6]. The difference between the probable prevalence of monogenic disease and the detection rate of routine testing supports the use of more comprehensive approaches such as WES. Proposed genetic tests in male infertility are summarised in Figure 1.

## 5. Methodological Principles of WES

WES involves the selective enrichment and sequencing of exons, which comprise the protein-coding regions of the genome. It is one of several genomic diagnostic approaches, alongside whole-genome sequencing (WGS) and targeted gene panels, each of which differs in scope, cost, and clinical application [13]. Although the exome accounts for only 1–2% of the genome, it contains most variants currently known to cause Mendelian disease. WES achieves a broad diagnostic yield of approximately 25–35% across diverse populations with suspected rare diseases [14], but the diagnostic yield specific to idiopathic non-obstructive azoospermia (NOA) after negative standard first-line testing (karyotype, AZF, and *CFTR* variants) typically rests between 10% and 15% [6,7].

WES serves as a middle ground between WGS and targeted gene panels (Table 2). It is less expensive and generally easier to analyse than WGS but does not comprehensively cover non-coding or regulatory regions and is less reliable for detecting large structural rearrangements. WGS therefore usually has a somewhat higher diagnostic yield, although the difference is modest in many clinical indications [14]. Targeted panels provide greater sequencing depth at selected loci, providing high coverage at a low cost, but they cannot identify candidate genes outside the predefined list. Thus, while suitable for well-defined phenotypes, their diagnostic value is restricted in highly heterogeneous conditions [13]. The clinical selection strategy increasingly favours WES for conditions such as male infertility; a genome-wide approach not only offers broad coverage but importantly allows for periodic data re-analysis as new gene-disease associations are rapidly established in the literature, thereby preventing diagnostic dead-ends [4,14,15].

In male infertility, WES combined with the analysis of single-nucleotide variants (SNVs) and copy-number variants (CNVs) has been proposed as a first-line strategy that could consolidate several separate genetic investigations. Conventional karyotyping would nevertheless remain necessary for the detection of balanced translocations and low-level mosaicism, which are not reliably identified by WES [7].

Once the physical sequencing is complete, the resulting data must be processed to identify causative mutations. The bioinformatic workflow converts the raw sequencing reads into a clinically relevant set of candidate variants. It includes alignment to the reference genome, calling of SNVs and small insertions or deletions, and sequential filtering based on population frequency, predicted functional effects, compatibility with the expected inheritance pattern, and the strength of the association between the affected gene and the patient’s phenotype.

**Figure 2:**
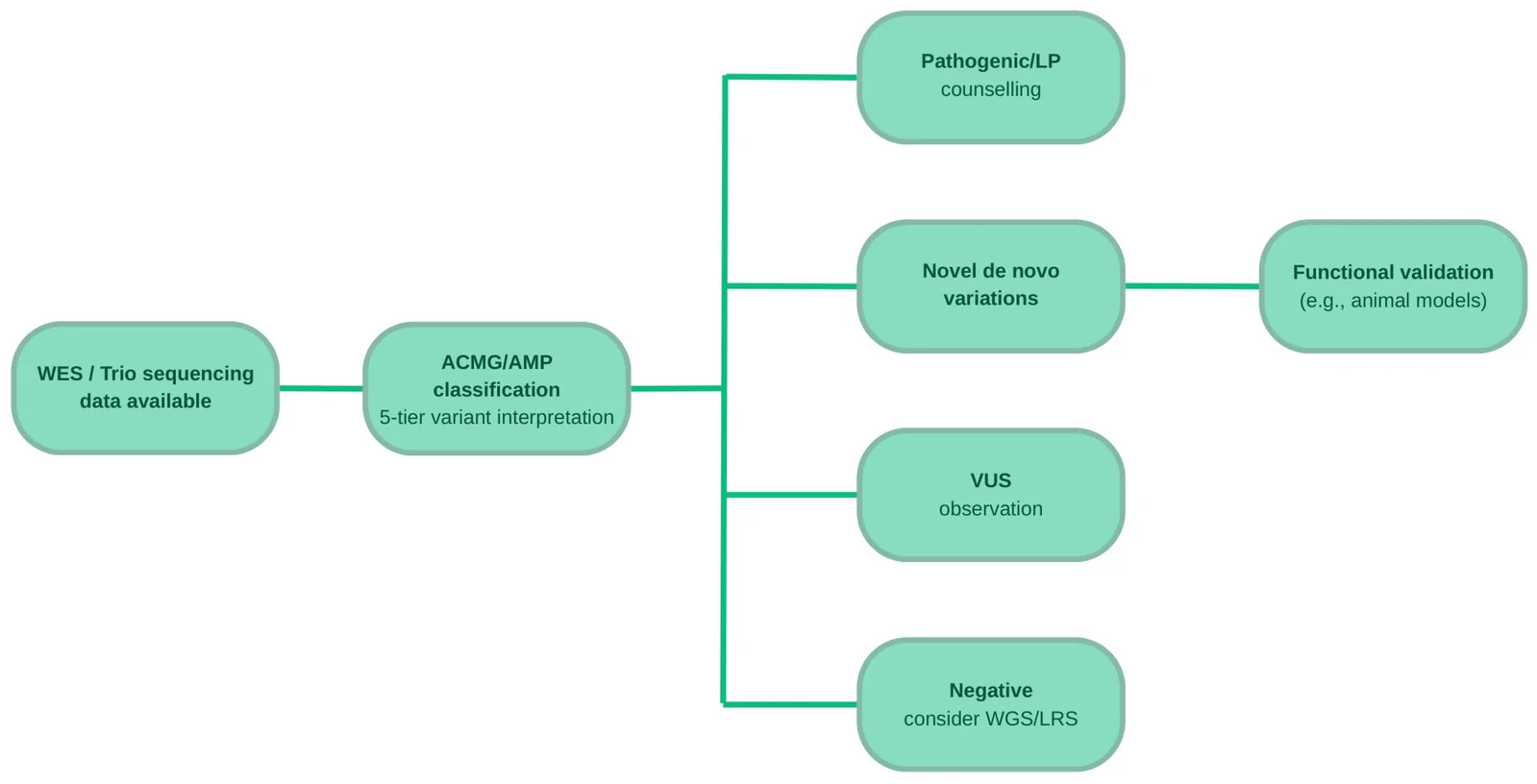
Proposed algorithm of male infertility diagnosis according to the type of variant. Variant classification based on American College of Medical Genetics and Genomics/Association for Molecular Pathology recommendations. Figure created using Canva Pro (Canva Pty Ltd., Sydney, Australia; https://www.canva.com).

Variants are subsequently classified according to the American College of Medical Genetics and Genomics/Association for Molecular Pathology (ACMG/AMP) framework as pathogenic, likely pathogenic, of uncertain significance, likely benign, or benign (Figure 2).

This classification system is routinely used in WES studies of male infertility. In an Italian cohort, pathogenic or likely pathogenic variants identified using this approach provided a molecular diagnosis in 12% of patients with previously idiopathic infertility [6]. In clinical practice, reports generally focus on pathogenic and likely pathogenic variants. Variants of uncertain significance (VUS), which account for a substantial proportion of WES findings, require additional evidence and should not independently guide clinical decisions. Consistent application of classification criteria is particularly important when evaluating variants in newly proposed candidate genes.

Study design also affects the types of variants that can be detected. Most WES studies of male infertility have used case–control designs in which variants identified in patients are compared with population databases or cohorts of fertile men. This approach is primarily suited to identifying recessive and X-linked variants. Trio sequencing, which analyses the patient and both parents, permits direct identification of *de novo* variants that are absent from parental genomes. A study of 185 trios comprising infertile men and their unaffected parents found significant enrichment of deleterious *de novo* variants in genes intolerant to loss of function. These findings strongly imply that *de novo* variants may account for a previously underestimated proportion of severe male infertility [15].

## 6. Genetic Basis of Infertility Revealed by WES

WES studies have contributed to the systematic identification and classification of genes associated with specific male-infertility phenotypes. A systematic review by the International Male Infertility Genomics Consortium (IMIGC) used an adapted semi-quantitative framework analogous to that applied by ClinGen to assess the strength of gene–disease associations. The review identified 120 genes with moderate, strong, or definitive evidence of association with 104 infertility phenotypes [8]. This framework helps distinguish genes with established clinical relevance from candidate genes supported only by isolated case reports, which is essential when designing diagnostic panels and interpreting WES findings [3].

The genes identified through WES can be considered in three principal phenotypic groups (Table 3). Clinical validity of these genes is further supported by corresponding functional validation in animal models (Table 4). The first includes quantitative disorders of spermatogenesis, particularly NOA and severe oligozoospermia. Genes associated with meiotic arrest, including *TEX11*, *STAG3*, *MEIOB*, and *M1AP*, are prominent in this group. Pathogenic variants in these genes may prevent the production of mature gametes despite preservation of the spermatogonial pool [3,16].

The second group comprises disorders of sperm motility. Multiple morphological abnormalities of the sperm flagella (MMAF) are among the best-characterised phenotypes in this category. Since *DNAH1* gene was first associated with MMAF, more than 40 genes encoding proteins involved in the axoneme, centrosome, and intraflagellar transport have been described. These include members of the *CFAP* family such as *CFAP43*, *CFAP44*, *CFAP69* and the genes *DNAH2*, *DNAH6*, and *DNAH17* [17].

The third group includes abnormalities of sperm-head morphology. These encompass well-characterised monogenic phenotypes, including globozoospermia, most associated with deletion of *DPY19L2* gene, and macrozoospermia, caused by recurrent variants in *AURKC* gene. WES has also identified candidate genes associated with the broader and genetically heterogeneous phenotype of teratozoospermia [18].

WES can additionally identify pre-testicular causes of infertility associated with dysfunction of the hypothalamic–pituitary–gonadal axis. Congenital hypogonadotropic hypogonadism (CHH), including normosmic CHH and Kallmann syndrome, results from GnRH deficiency and is one of the few genetically determined causes of male infertility for which causal hormonal treatment is available. More than 40 genes have been associated with CHH, including *ANOS1*, *GNRHR*, *FGFR1*, *KISS1R*, and *TAC3*. Oligogenic inheritance is relatively common: in some patients, the phenotype results from variants in two or more genes. WES is well suited to detecting this type of genetic architecture because its analysis is not restricted to a small predefined panel [19].

However, these clinical phenotypes frequently overlap in practice. Some genes are associated with combined abnormalities of sperm number, motility, and morphology, reflecting molecular pathways involved in multiple stages of spermatogenesis and spermiogenesis [4].

**Table 3:** Clinical validity of candidate gene–disease relationships (GDRs) for male-infertility phenotypes, comparing the systematic reviews of Houston et al. [8] and Zhao et al. [20]. Score/classification pairs follow the five-tier system of Smith et al. [21]: no evidence (<3), limited (3–8), moderate (9–12), strong (13–15), definitive (>15).

Gene	Location	OMIM Phenotype ID	Inheritance	Houston et al. (Score/Classification)	Zhao et al., (Score/Classification)	gnomAD Constraint (pLI/LOEUF)
Meiotic arrest/non-obstructive azoospermia (NOA)						
*TEX11* ^a^	Xq13.1	309120	XL	16/Definitive	16/Definitive (no change)	pLI–1/LOEUF–0.16
*STAG3*	7q22.1	619672	AR	11.5/Moderate	14/Strong (increased)	pLI–0/LOEUF–0.74
*SYCE1*	10q26.3	616950	AR	8/Limited	10/Moderate (increased)	pLI–0/LOEUF–0.88
*SYCP3*	12q23.2	270960	AD	14/Strong	Not re-evaluated (absent from Table 3)	pLI–0/LOEUF–1.03
*REC8*	14q12	Not established	Not established	Not assessed	Not assessed	pLI–0/LOEUF–0.69
*MEIOB*	16p13.3	617706	AR	8/Limited	14/Strong (increased)	pLI–0/LOEUF–1.19
*MEI1*	22q13.2	Not established	AR	13/Strong	Not re-evaluated (absent from Table 3)	pLI–0/LOEUF–0.83
*M1AP*	2p13.1	619108	AR	12/Moderate	14/Strong (increased)	pLI–0/LOEUF–1.00
Multiple morphological abnormalities of the sperm flagella (MMAF)						
*CFAP43*	10q25.1	617592	AR	17/Definitive	17/Definitive (no change)	pLI–0/LOEUF–0.81
*CFAP44*	3q13.2	617593	AR	17/Definitive	17/Definitive (no change)	pLI–0/LOEUF–0.72
*CFAP69*	7q21.13	617959	AR	13/Strong	13/Strong (no change)	pLI–0/LOEUF–0.89
*DNAH1*	3p21.1	617576	AR	17/Definitive	18/Definitive (no change in tier)	pLI–0/LOEUF–0.74
*DNAH8*	6p21.2	619095	AR	Not yet reported	18/Definitive (new gene)	pLI–0/LOEUF–0.74
*DNAH17*	17q25.3	618643	AR	15/Strong	18/Definitive (increased)	pLI–0/LOEUF–0.84
Globozoospermia						
*DPY19L2*	12q14.2	613958	AR	16/Definitive	16/Definitive (no change)	pLI–1/LOEUF–0.48
*SPATA16*	3q26.31	102530	AR	Not listed (≤Limited)	Not addressed	pLI–0/LOEUF–0.75
*ZPBP1*	7p12	619799	AR	Not listed (≤Limited)	Not addressed	pLI–0/LOEUF–0.81
Macrozoospermia						
*AURKC*	19q13.43	243060	AR	17/Definitive	17/Definitive (no change)	pLI–0.01/LOEUF–0.79
Acephalic spermatozoa						
*SUN5*	20q11.21	617187	AR	16.75/Definitive	17/Definitive (no change)	pLI–0/LOEUF–1.05
*PMFBP1*	16q22.2	618112	AR	14/Strong	15/Strong (no change in tier)	pLI–0/LOEUF–1.10
Fertilization failure						
*PLCZ1*	12p12.3	617214	AR	16/Definitive	16/Definitive (no change)	pLI–0/LOEUF–1.03

^a^ Houston et al. [8] give this gene’s location as Xp11 in the published text—an apparent error, since Xq13.1 is the established cytogenetic location and is the value used by Zhao et al. [20] and reproduced here.

**Table 4:** Animal-model support for each gene–disease relationship, independently verified against primary literature.

Gene	Animal Model	Evidence	Ref.
*TEX11*	Confirmed	Tex11-null male mice: chromosomal asynapsis, reduced crossover formation, MI nondisjunction, infertility—closely mirrors human meiotic-arrest phenotype.	[22]
*STAG3*	Confirmed	Stag3-null mice (both sexes): zygotene-like arrest, failure of axial-element/synaptonemal-complex formation, sterility. Independently generated in two labs.	[23,24]
*SYCE1*	Confirmed	Syce1-null mice: failure of homologous synapsis, pre-pachytene arrest with apoptosis, complete infertility in both sexes.	[25]
*SYCP3*	Confirmed	Sycp3-null males: zygotene arrest, complete azoospermia. Females are subfertile (not infertile)—oocyte aneuploidy/embryo loss, not gametogenic arrest.	[26]
*REC8*	Confirmed	Rec8-null mice (both sexes) are sterile; in the absence of REC8, synapsis occurs between sister chromatids rather than homologs. Original mutant positionally cloned by a second group.	[27,28]
*MEIOB*	Confirmed	Meiob-null mice (both sexes): zygotene/pachytene-like arrest, impaired DSB repair and synapsis. Independently replicated by a second group the same year.	[29,30]
*MEI1*	Confirmed	mei1 mutant mice (both sexes): defective chromosome synapsis, no RAD51 loading onto meiotic chromosomes despite DSB formation. Positionally cloned in a follow-up paper.	[31,32]
*M1AP*	Confirmed	M1ap-deficient males: most cells reach metaphase I before arresting (abnormal synapsis, no crossover foci); severe oligozoospermia and infertility. Females fertile with normal ovaries.	[33]
*CFAP43*	Confirmed	Cfap43-knockout males: MMAF, disorganized axoneme, absent central-pair complex—mirrors human ultrastructure. Independently replicated by a second group.	[34,35]
*CFAP44*	Confirmed	Cfap44-knockout males: MMAF, generated and characterised alongside the CFAP43 knockout in the same studies.	[34,35]
*CFAP69*	Confirmed	Cfap69-knockout males: MMAF phenotype recapitulating the human patients’ flagellar defects.	[36]
*DNAH1*	Confirmed	Dnah1 (Mdhc7)-null males: severely reduced sperm motility/straight-line velocity, sperm cannot reach the oviduct, infertile. Predates the human MMAF association by over a decade.	[37,38]
*DNAH8*	Confirmed	Dnah8-knockout males: full MMAF phenotype and sterility; ICSI using knockout sperm produced healthy offspring.	[39]
*DNAH17*	Confirmed	Dnah17-null mice: MMAF with completely disorganized axonemal structure, closely matching patients’ ultrastructure.	[40]
*DPY19L2*	Confirmed	Dpy19l2-null males: ~100% globozoospermic, acrosomeless sperm, complete infertility	[41]
*SPATA16*	Confirmed	CRISPR/Cas9 Spata16 mouse models: impaired sperm/acrosome formation consistent with human globozoospermia.	[42]
*ZPBP1*	Confirmed	Zpbp1-null males: sterile, round-headed sperm morphology, acrosome fragmentation and Sertoli–spermatid junction disruption.	[43]
*AURKC*	Partial	Aurora-C-knockout mice have compromised (not absent) fertility, with heterogeneous chromatin condensation, loose acrosomes and blunted (not enlarged/multi-tailed) sperm heads. This is a related meiotic/spermiogenesis defect, not a clean phenocopy of the human tetraploid, macrocephalic, multiflagellate phenotype.	[44]
*SUN5*	Confirmed	Sun5-null mice (two independently generated lines): the head–tail coupling apparatus detaches from the nucleus during spermatid elongation, producing acephalic/pseudo-globozoospermic sperm.	[45,46]
*PMFBP1*	Confirmed	Pmfbp1-knockout males: acephalic spermatozoa via disrupted PMFBP1–SUN5–SPATA6 head–tail coupling; ICSI restored fertility.	[47]
*PLCZ1*	Confirmed	Plcz1-knockout sperm fail to trigger normal Ca^2+^ oscillations in oocytes, causing oocyte-activation failure and/or polyspermy; generated independently by two groups the same year with concordant conclusions.	[48,49]

## 7. Diagnostic Yield of WES Across Different Phenotypes

The diagnostic yield of WES in male infertility depends on the phenotype, cohort size and selection criteria, and the bioinformatic and interpretative methods used. When defining the cohort strictly as patients with true idiopathic NOA who remain undiagnosed after standard testing, the pooled diagnostic yield of WES is approximately 15% [50]. However, substantially higher detection rates (ranging from 27% to over 50%) have been reported for rare, highly selected qualitative phenotypes, particularly in cohorts enriched for consanguinity [4,51].

NOA is the most extensively studied phenotype. A meta-analysis of nine studies including 1728 men with idiopathic NOA estimated a pooled diagnostic yield of 15% (95% CI: 10–20%). Because of considerable methodological heterogeneity, the authors cautioned that this estimate represents an average across different clinical settings rather than a precise value applicable to every population [50]. The largest individual WES cohort in NOA was reported by the international GEMINI consortium and included more than 1000 men with clinically confirmed disease. A probable recessive cause was identified in approximately 20% of participants. Twenty-one genes received additional support through a two-stage variant-burden analysis involving an independent cohort of more than 2000 cases and nearly 11,600 fertile controls [51].

Similar yields have been reported for other quantitative spermatogenic disorders. In a cohort of 521 men with idiopathic spermatogenic failure analysed using a panel of 638 candidate genes, a molecular diagnosis was established in 12% of participants. The yield did not differ significantly between men with azoospermia and those with oligozoospermia [52]. A diagnostic yield of 12% was also reported in the Italian cohort described above [6]. Together, these studies suggest that the yield in severe quantitative disorders of spermatogenesis is commonly approximately 10–15%.

Estimates are more variable for qualitative sperm abnormalities. WES identified a molecular diagnosis in 14.6% of patients with unexplained heterogeneous abnormalities of the sperm head. In small, highly selected cohorts with acephalic spermatozoa syndrome, reported yields ranged from 27.3% to 58.3%. Studies of asthenozoospermia and MMAF have likewise produced variable results; however, higher diagnostic yields in MMAF are often driven by cohorts enriched for consanguinity and the presence of highly recurrent variants within specific gene families, such as *DNAH* and *CFAP* [4].

Diagnostic yield varies considerably between centres primarily due to differing eligibility criteria, with some cohorts restricted to strictly defined, true idiopathic infertility, while others include broader, more heterogeneous populations (Table 5). Studies also differ in the number and selection of analysed genes, from limited sets of well-established genes to candidate panels containing several hundred genes. In rare qualitative phenotypes, small cohorts make diagnostic-yield estimates particularly sensitive to individual cases and limit their generalisability [50].

## 8. Clinical Significance of an Identified Molecular Diagnosis

Identifying the molecular cause of infertility directly alters clinical management by providing clear prognostic insights and guiding therapeutic interventions [53]. Establishing a precise diagnosis enables informative genetic counselling, as patients carrying pathogenic variants face a risk of transmitting male infertility to their potential progeny if assisted reproduction is successful [54].

A molecular diagnosis also serves as a powerful prognostic tool for predicting the likelihood of successful sperm retrieval by testicular sperm extraction (TESE) [50,53]. Pathogenic variants in meiotic genes are associated with extremely low retrieval rates. For instance, reported TESE success rates are as low as 8.3% in cases of Sertoli cell-only syndrome and 8.7% in meiotic arrest [50]. Incorporating well-validated genes into pre-TESE prognostic assessments adds substantial clinical utility, as men with specific genetic defects in meiotic pathways are theoretically unlikely to yield viable sperm [50]. Notable genes with a defined biological basis for poor TESE outcomes include:

*TEX11*: Mutations in this gene impair the assembly and function of the synaptonemal complex. This causes severe defects in chromosome synapsis during the pachytene stage, resulting in meiotic arrest and predominantly negative TESE outcomes [50].

*STAG3*: This gene encodes a component of the meiosis-specific cohesin complex that is essential for maintaining chromatid cohesion, DNA repair, and synapsis between homologous chromosomes [54].

*MSH4*: This gene plays a critical biological role in the reciprocal recombination and segregation of homologous chromosomes during meiosis [54].

*TERB1*, *MEIOB*, and *SPO11*: These are critical meiotic genes; loss-of-function mutations disrupting these genes have been strongly linked to meiotic arrest and uniformly negative TESE outcomes in studied cohorts [53].

Histopathological evidence of meiotic arrest strongly correlates with variants in these meiosis-involved genes, reinforcing the diagnostic and prognostic relevance of these findings [53]. Identifying a relevant variant before surgery can therefore help patients avoid an invasive procedure when the probability of success is minimal [50,54]. This approach not only spares patients from futile surgeries but also reduces their exposure to potential long-term adverse effects of TESE, such as androgen deficiency. Ultimately, a known genetic prognosis allows for more targeted clinical decision-making, including the potential for earlier inclusion in donor programs [53,54].

## 9. Challenges and Limitations

While WES offers undeniable diagnostic value, its routine use in male infertility remains limited by methodological, organisational, and ethical challenges.

The sheer volume of VUS remains the primary interpretative bottleneck. In the Italian cohort described above, only 12 of 189 classified variants were considered pathogenic or likely pathogenic, whereas most of the remaining variants were classified as VUS [6]. This imbalance reflects the relatively recent development of research into monogenic male infertility and the limited functional validation of newly identified variants. To cross this functional validation gap, the gold standard remains a mouse knockout model that faithfully reproduces the predicted infertility or spermatogenic phenotype; genes with strong human statistical evidence but lacking animal model support frequently remain classified as candidates [8,16]. The field also increasingly employs a reverse logic: when a mouse knockout causes infertility but lacks a human report, it becomes a strong candidate for a novel human male infertility gene [16]. Additionally, variant interpretation in male infertility requires adaptation of standard ACMG/AMP rules. Computational evidence leans heavily on conservation and in silico predictors, while loss-of-function (LoF) intolerance metrics, such as the pLI score, significantly strengthen a LoF claim [15]. A variant’s reporting is ultimately dictated by the gene’s disease-validity tier, underscoring the importance of IMIGC classifications [3,8]. VUS should therefore not be used independently to guide decisions such as abandoning TESE or changing the infertility-treatment strategy. Their uncertain interpretation limits the clinical utility of some WES findings and may require re-evaluation as new evidence becomes available.

Population ancestry represents an additional challenge in genomic variant interpretation. Reference population databases, including gnomAD, provide essential allele-frequency information for variant classification; however, the representation of global populations remains uneven. Consequently, the absence or low frequency of a variant in a reference database may be difficult to interpret when the relevant population is underrepresented, potentially contributing to uncertainty in variant classification. Ancestry-aware interpretation and consideration of population-specific allele frequencies are therefore important when applying population-based ACMG/AMP criteria. Expanding the representation of diverse populations in genomic reference databases and male infertility cohorts will be essential for improving the accuracy and equity of genetic diagnosis [55].

Beyond these interpretative challenges, there are technological limitations to standard short-read WES when applied to NOA. Because WES relies almost exclusively on blood-derived DNA, it is fundamentally blind to gonadal or testicular mosaicism, which may drive spermatogenic failure in some men [13]. Short-read WES also struggles significantly with the detection of complex structural variants and copy-number changes, and it cannot evaluate deep-intronic regulatory or non-coding variants that might impact gene expression [4,13]. While the promise of an “all-in-one” genomic test is appealing, standard short-read WES cannot reliably call common structural rearrangements such as partial AZFc microdeletions (e.g., *gr*/*gr* or *b2*/*b3* deletions); thus, dedicated calling algorithms, sequence-tagged site PCR (STS-PCR), or digital droplet PCR remain essential alongside WES [7,56].

The absence of a standardised set of genes for WES analysis presents a further challenge. Despite the work of initiatives such as the IMIGC and the adaptation of ClinGen-based methods [8], no uniform and widely accepted clinical gene set for male infertility has been established [54]. Centres differ in the number and choice of genes analysed, contributing to variation in reported diagnostic yields.

Cost and access also limit implementation. Although sequencing costs continue to decrease, and cost-effectiveness studies in other rare diseases suggest that WES may reduce healthcare expenditure per additional diagnosis [57], access remains geographically uneven and dependent on national reimbursement policies [58]. Some centres also lack the bioinformatic infrastructure and clinical genetics expertise needed to interpret results in a condition with substantial phenotypic heterogeneity.

Ethical concerns arise from the potential identification of secondary findings unrelated to infertility but relevant to the patient’s health, including variants associated with cancer predisposition [59]. This issue may be particularly relevant given reports of increased cancer risk among infertile men [2,3,6]. Pre-test genetic counselling should address the possibility of secondary findings and establish the patient’s preferences regarding disclosure. Consequently, pre-test counselling must be exhaustive, adding complexity to the informed consent process.

## 10. Future Perspectives

The next era of andrological genetics will inevitably rely on converging multi-omics data and falling sequencing costs.

One area of development is the use of “all-in-one” strategies that combine SNV and CNV analysis within a single WES assay. Such strategies may consolidate much of the current first-line genetic pathway, including testing for AZF microdeletions and *CFTR* variants, while reducing the time and cost associated with separate investigations. Conventional karyotyping would still be required to detect balanced translocations and low-level mosaicism [7].

WES may also be integrated with transcriptomic, epigenomic, and proteomic data. Systematic reviews indicate that different omics approaches identify largely distinct and only partially overlapping molecular factors associated with infertility. Analysis of coding variants alone is therefore unlikely to capture the full spectrum of causes of spermatogenic failure. Combining WES with transcriptomic data from testicular biopsies or epigenetic profiles of sperm may help resolve cases that remain unexplained after exome sequencing [60,61].

WGS and long-read sequencing (LRS) may further expand diagnostic capability. By covering non-coding and regulatory regions, WGS could eventually serve as a comprehensive first-line test encompassing abnormalities currently assessed by karyotyping, AZF microdeletion analysis, and WES [62]. LRS enables the analysis of long, continuous DNA fragments and may improve the detection of complex structural rearrangements, discrimination between highly homologous genes and pseudogenes, and determination of the cis or trans configuration of variants without parental samples. In an early application relevant to infertile men, LRS increased molecular detection by more than 30% relative to a standard NGS panel within the group of variants analysed [63]. Although cost and bioinformatic complexity currently limit routine use, LRS may complement WES when a structural cause is suspected or when short-read sequencing produces an ambiguous result.

## 11. Conclusions

Whole-exome sequencing is an effective tool for investigating the molecular basis of idiopathic male infertility and addresses part of the diagnostic gap left by standard genetic testing. Although its diagnostic yield varies according to phenotype and study methodology, a confirmed molecular diagnosis can improve genetic counselling, inform assessment of risk to offspring, help predict the outcome of TESE or micro-TESE, and support treatment planning using assisted reproductive technologies.

Important limitations remain, including the high frequency of VUS, the lack of standardised gene sets and analytical pipelines, unequal access to testing, and ethical concerns related to secondary findings. Future diagnostic strategies are likely to combine WES-based SNV and CNV analysis with other omics approaches, WGS, and LRS. With further validation and standardisation, comprehensive genomic profiling may become a part of routine diagnostic pathways for male infertility.

## Figures and Tables

**Table 1:** Indications, utility and limitations of standard first-line genetic tests vs. WES in male infertility.

Test	AUA/ASRM Indication	Scope	Diagnostic Yield	Key Limitation
Karyotype	Azoospermia or sperm concentration < 5 million/mL, with elevated FSH concentration, testicular atrophy, or a diagnosed spermatogenic disorder	Numerical/structural chromosomal aberrations only	~15% in azoospermia (mostly Klinefelter syndrome)	Cannot detect single-gene causes
AZF microdeletion analysis	Azoospermia or sperm concentration ≤ 1 million/mL	Three defined Yq regions (AZF a/b/c)	8–12% in NOA; 3–7% in severe oligozoospermia	Limited to Y-chromosome long-arm deletions
*CFTR* gene testing	Congenital absence of the vas deferens or idiopathic obstructive azoospermia	Single gene	60–90% of CBAVD cases	Relevant only to CBAVD-related infertility
Combined standard panel	Severe oligozoospermia/azoospermia	Numerical/structural chromosomal aberrations, three AZF regions, and the *CFTR* gene	4–9.2% of all cases; up to ~25% in NOA	Explains only a minority of cases
WES	Idiopathic infertility after negative standard work-up	~95% of coding regions, all genes simultaneously	~10–20% overall (phenotype-dependent)	High VUS rate; misses balanced translocations/mosaicism

**Table 2:** Comparison of targeted genetic panels testing vs. WES and WGS.

Feature	Targeted Panels	WES	WGS
Coverage	Known loci only, highest depth	~1–2% of genome, ~95% of coding regions	Whole genome incl. non-coding/regulatory regions
Novel candidate-gene discovery	No	Yes	Yes
Structural variant/CNV detection	Limited	Possible with dedicated CNV analysis	Better than WES
Cost/turnaround	Lowest	Intermediate	Higher, slower analysis
Typical diagnostic yield	Depth-dependent, variable	~25–35%	Slightly higher than WES, usually modestly

**Table 5:** Diagnostic yield of WES across infertility phenotypes.

Phenotype	Cohort	Diagnostic Yield
Idiopathic NOA (pooled)	1728 men, 9 studies	15% (95% CI: 10–20%)
NOA (GEMINI consortium)	>1000 men	~20% probable recessive cause
Idiopathic spermatogenic failure	521 men, 638-gene panel	12%
Idiopathic infertility (Italian cohort)	99 men	12%
Unexplained sperm-head abnormalities	82 men	14.6%
Acephalic spermatozoa syndrome	Small, selected cohorts	27.3–58.3%

## Data Availability

No new data were created or analyzed in this study. Data sharing is not applicable to this article.

## Supplementary Materials

The following supporting information can be downloaded at: https://www.mdpi.com/article/10.3390/jcm15187281/s1, Document S1: SANRA (Scale for the Assessment of Narrative Review Articles) checklist. Reference [64] is cited in the Supplementary Materials.

## Abbreviations

The following abbreviations are used in this manuscript:

ACMG/AMP	American College of Medical Genetics and Genomics/Association for Molecular Pathology
AD	Autosomal dominant
AR	Autosomal recessive
ART	Assisted reproductive technologies
AUA/ASRM	American Urological Association/American Society for Reproductive Medicine
AZF	Azoospermia factor
CBAVD	Congenital bilateral absence of the vas deferens
CHH	Congenital hypogonadotropic hypogonadism
CI	Confidence interval
ClinGen	Clinical Genome Resource
CNV	Copy-number variant
CRISPR/Cas9	Clustered regularly interspaced short palindromic repeats/CRISPR-associated protein 9
DNA	Deoxyribonucleic acid
DSB	DNA double-strand break
FSH	Follicle-stimulating hormone
GDR	Gene-disease relationship
gnomAD	Genome Aggregation Database
GnRH	Gonadotropin-releasing hormone
ICSI	Intracytoplasmic sperm injection
IMIGC	International Male Infertility Genomics Consortium
LOEUF	Loss-of-function observed/expected upper bound fraction
LRS	Long-read sequencing
Micro-TESE	Microdissection testicular sperm extraction
MMAF	Multiple morphological abnormalities of the sperm flagella
NGS	Next-generation sequencing
NOA	Non-obstructive azoospermia
OA	Obstructive azoospermia
OMIM	Online Mendelian Inheritance in Man
PCR	Polymerase chain reaction
pLI	Probability of loss-of-function intolerance
SNV	Single-nucleotide variant
STS-PCR	Sequence-tagged site polymerase chain reaction
TESE	Testicular sperm extraction
VUS	Variant of uncertain significance
WES	Whole-exome sequencing
WGS	Whole-genome sequencing
XL	X-linked
